# Dissecting the essential role of N-glycosylation in catalytic performance of xanthan lyase

**DOI:** 10.1186/s40643-022-00620-5

**Published:** 2022-12-16

**Authors:** Jingjing Zhao, Qian Wang, Xin Ni, Shaonian Shen, Chenchen Nan, Xianzhen Li, Xiaoyi Chen, Fan Yang

**Affiliations:** 1 School of Biological Engineering, Dalian Polytechnic University, Ganjingziqu, 116034 Dalian People’s Republic of China; 2 Division of Biotechnology, Dalian Institute of Chemical Physics, Chinese Academy of Sciences, Dalian, 116023 People’s Republic of China

**Keywords:** Xanthan lyase, N-Glycosylation, Site-directed mutagenesis, Structure regulation, Catalytic properties

## Abstract

Modified xanthan produced by xanthan lyase has broad application prospects in the food industry. However, the catalytic performance of xanthan lyase still needs to be improved through rational design. To address this problem, in this work, the glycosylation and its influences on the catalytic performance of a xanthan lyase (*Ec*Xly), which was heterologously expressed in *Escherichia coli*, were reported. Liquid chromatography coupled to tandem mass spectrometry analysis revealed that the N599 site of *Ec*Xly was modified by a single N-glycan chain. Based on sequence alignment and three-dimensional structure prediction, it could be deduced that the N599 site was located in the catalytic domain of *Ec*Xly and in close proximity to the catalytic residues. After site-directed mutagenesis of N599 with alanine, aspartic acid and glycine, respectively, the *Ec*Xly and its mutants were characterized and compared. The results demonstrated that elimination of the N-glycosylation had diminished the specific activity, pH stability, and substrate affinity of *Ec*Xly. Fluorescence spectra further revealed that the glycosylation could significantly affect the overall tertiary structure of *Ec*Xly. Therefore, in prokaryotic hosts, the N-glycosylation could influence the catalytic performance of the enzyme by changing its structure. To the best of our knowledge, this is the first report about the post-translational modification of xanthan lyase in prokaryotes. Overall, our work enriched research on the role of glycan chains in the functional performance of proteins expressed in prokaryotes and should be valuable for the rational design of xanthan lyase to produce modified xanthan for industrial application.

**Graphical Abstract:**

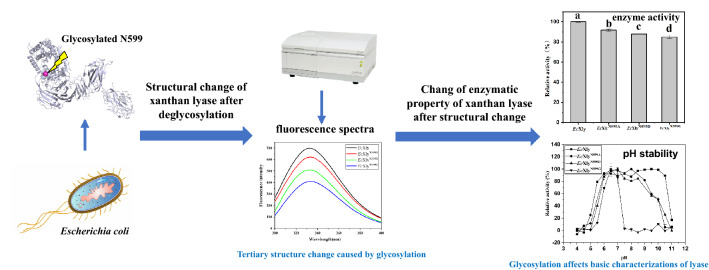

**Supplementary Information:**

The online version contains supplementary material available at 10.1186/s40643-022-00620-5.

## Introduction

Xanthan is an extracellular water-soluble polysaccharide secreted by the pathogenic bacterium *Xanthomonas campestris* (Patel et al. 2020) The cellulosic backbone of xanthan consists of a cellobiose unit, and the branching trisaccharide (β-D-Man*p*-(1 → 4)β-D-GlcUA*p*-(1 → 2)α-D-Man) is α-1,3-linked to alternating backbone glucose units (Jansson et al. 1975). The complex structure endows xanthan with special rheological properties, which makes xanthan a widely utilized thickener, gelling agent and stabilizer in food additives industry (Habibi and Khosravi-Darani. 2017). It is believed that molecular modification could endow xanthan with novel physicochemical and physiological functions, which will expand the scope of xanthan application in the food section. Previous reports demonstrated that modifications of side chain have great impacts on xanthan’s rheological properties, solubility and dispersion (Riaz et al. 2021). Notably, xanthan with truncated side chain is less susceptible to high temperature (De Sousa et al. 2019). Some *Xanthomonas campestris* strains have been genetically engineered to produce xanthan with modified side chains, but the yield of which cannot meet the industrial demands (Tait et al. 1989). Therefore, enzymatic modification of xanthan is considered to be an alternative strategy.

Xanthan lyase belongs to the polysaccharide lyase 8 (PL8) family and plays an important role in the xanthan-degrading process (Jensen et al. 2019). It can cleavage the α-1,2 glycosidic bonds between terminal mannose and glucuronic acid of the branching trisaccharide through β-elimination reaction, producing mutant xanthan with low viscosity (Hassler and Doherty. 1990). Previous works revealed that xanthan lyase expressed in *Escherichia coli* hosts exhibited a better thermal stability compared with the native xanthan lyase from *Paenibacillus alginolyticus* XL-1 (Ruijssenaars et al. 2000, 1999). Other researches also proved that the specific activity of *E. coli*-expressed xanthan lyase was significantly higher than that of the native xanthan lyase from *Bacillus* sp. GL1 (Hashimoto et al. 2001, 1998). All these findings supported that xanthan lyases produced from different hosts possess different properties, which could be attributed to many factors, e.g., the discrepancy in post-translational modifications of the enzyme.

As an important post-translational modification in eukaryotes, glycosylation can affect protein properties in many aspects, such as proper folding, solubility, thermal stability, catalytic activity and proteolysis (Varki 2017). Besides eukaryotes, glycosylation has also been found in bacterium such as *Campylobacter jejuni*, *Neisseria meningitides*, *Pseudomonas aeruginosa* and *E.coli* (Balonova et al. 2009; Wacker et al. 2002). The N-glycosylation process involves the transportation of heptasaccharide from the undecyl pyrophosphate donor to the asparagine side chain of the bacterial periplasmic membrane (Nothaft and Szymanski. 2010). It is generally believed that prokaryotic N-glycosylation usually occurs on the protein sequence N-X-S/T, where X can be any amino acid but not proline. As one of the examples, the strain *Campylobacter jejuni* possesses a *pgl* (protein glycosylation) locus-dependent general N-glycosylation system of proteins. (Nita-Lazar et al. 2005). To date, for eukaryotes, the effect of glycosylation on enzyme properties has been clearly demonstrated. However, little information is available for the role of glycan chains in functional performance of proteins expressed in prokaryotes.

At present, to meet the demand for the industrial production of modified xanthan, the catalytic performance of xanthan lyase still needs to be improved through the rational design. To address this issue, our work investigated the influence of glycan chains on the catalytic performance of a xanthan lyase heterologously expressed in *E.coli*, and the key N-glycosylation site was determined by liquid chromatography coupled to tandem mass spectrometry. Then, based on the site-directed mutagenesis, the effect of glycosylation on enzymatic properties was illuminated. Our findings should make an important contribution to the rational design of xanthan lyase and industrial production of modified xanthan, which might have wider applications as a novel thickening, stabilizing, or suspending agent used in food, pharmaceutical, cosmetic, and petroleum extraction aspects.

## Materials and methods

### Strains and culture conditions

*Microbacterium* sp. XT11 was cultured at 30 °C in xanthan medium (3 g xanthan, 0.5 g glucose, 3 g yeast extract, 0.025 g MgSO_4_·7H_2_O, 0.05 g K_2_HPO_4_, 0.8 g NaCl and 0.7 g KNO_3_ dissolved in 1 L deionized water, pH 7.0). *Escherichia coli* strains were cultivated at 37 °C in 1 L Luria–Bertani (LB) medium (10 g NaCl, 5 g yeast extract, and 10 g tryptone, pH 7.0), and 100 μg/mL ampicillin or 30 μg/mL kanamycin was added if necessary.

### Cloning, expression and purification of the gene encoding xanthan lyase

The xanthan lyase expressed in *E. coli* (*Ec*Xly) was obtained according to the following method. The gene encoding xanthan lyase was cloned from *Microbacterium* sp. XT11 (Yang et al. 2014) genome by PCR amplification using the primes Fwd and Rev (Additional file 1: Table S1). The amplification products were ligated to the vector pET-32a by restriction-free cloning method (RF-cloning) (Van den Ent et al. 2006). The methylated plasmid pET-32a among the PCR mixtures was digested with 0.5 μL of *Dpn*I at 37 °C for 1 h, and the products were then transducted into *E. coli* DH5α. The resulted plasmid pET-*EcXly* verified by DNA sequencing was transformed into *E. coli* Rosetta-gami (DE3) pLysS. The recombinants were selected on LB plates containing 100 μg/mL ampicillin and 30 μg/mL kanamycin.

For protein expression, *E. coli* Rosetta-gami (DE3) pLysS containing plasmid pET-*EcXly* was first cultured in LB low salt medium containing 100 μg/mL ampicillin and 30 μg/mL kanamycin at 37 °C. When the OD_600_ reached 0.6, a final concentration of 1 mM IPTG was added and the culture was further grown at 16 °C for 16–20 h. The cells were harvested by centrifugation at 8000 g for 5 min. The precipitate was collected, resuspended in NaH_2_PO_4_–NaCl buffer, and ultrasonicated. The debris was removed by centrifuging at 12,000 g for 20 min. The supernatant was used as the crude extract for enzyme purification by Ni–NTA affinity chromatography due to the ability of the protein with His-tag to bind to nickel (Crowe et al. 1994). The purity of the fraction was confirmed by SDS-PAGE. The protein concentration was measured using Bio-Rad protein assay kit (Bio-Rad, USA). The xanthan lyase derived from *Microbacterium* sp. XT11 (*Mi*Xly) was purified by ammonium sulfate fractionation, hydrophobic interaction chromatography and anion exchange chromatography in sequence according to the procedure of Yang et al. (2014).

### Analysis of glycosylation site

**Fig. 1:**
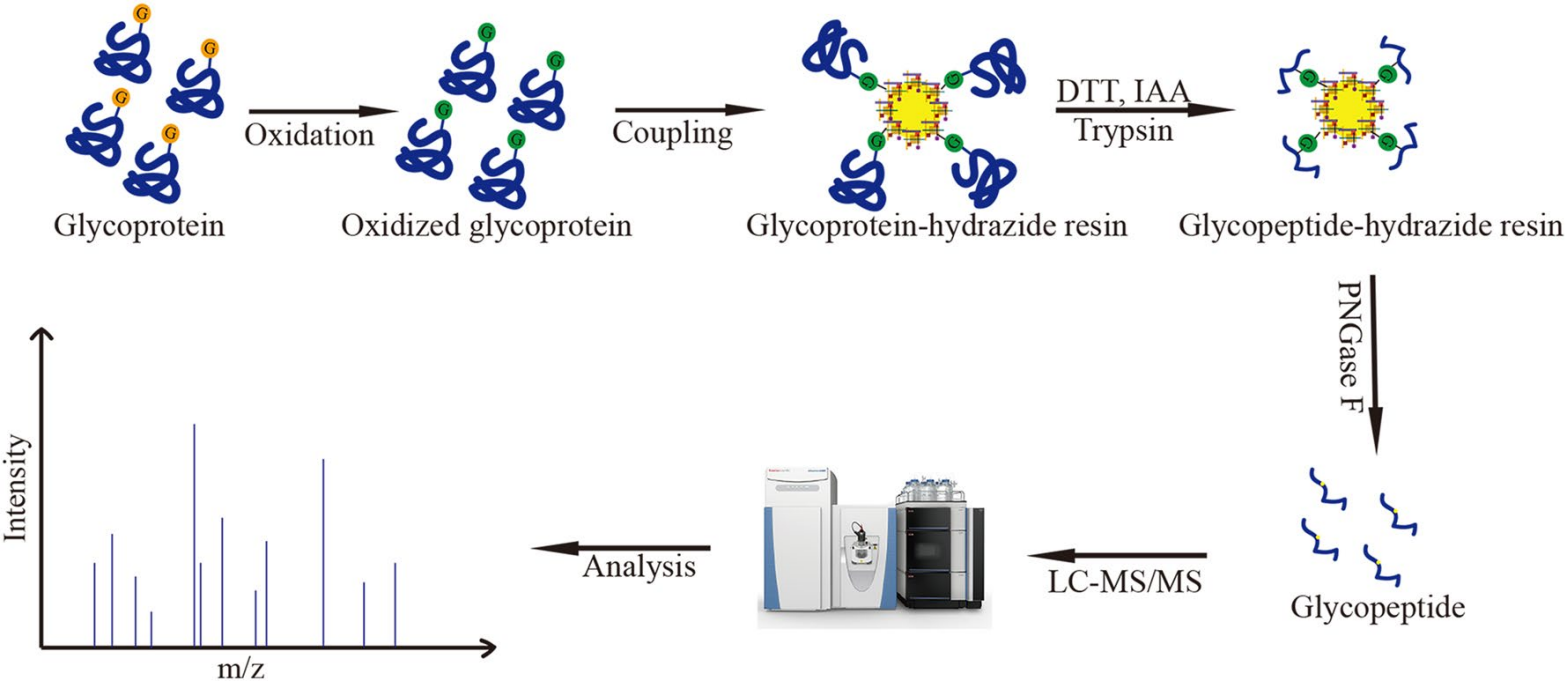
The schematic workflow of glycosylation analysis using LC–MS/MS

Based on specific capture of glycoproteins by hydrazide resin, the N-glycosylation sites were analyzed and identified using the LC–MS/MS (Thermo Scientific, MA, USA) (Zhang et al. 2003) (Fig. 1). 1 mg *Ec*Xly were dissolved in coupling buffer (100 mM NaAc, 150 mM NaCl), and 15 mM NaIO_4_ was added to oxidize the proteins for 1 h. NaIO_4_ was then removed using the ultrafiltration tube, and the proteins were coupled with hydrazide resin (Bio-Rad, USA) at room temperature for 10–24 h. To remove the nonglycoproteins, the sample was washed six times using an equal volume of buffer. The proteins were reduced by adding 20 mM dithiothreitol at 60 °C for 1 h and subsequently alkylated by the addition of 20 mM iodoacetamide in the dark for 40 min. The trypsin was used to hydrolyze proteins into peptides, and the PNGase F was used to release the enriched glycopeptides from hydrazide resin. Finally, the released peptides were desalinated and resuspended in an appropriate amount of 0.1% formic acid for further LC–MS/MS analysis. The peptides of *Mi*Xly and the mutants of *Ec*Xly were obtained by in-gel digestion and used for LC–MS/MS analysis (De Godoy et al 2006). The acquired MS data were analyzed using Proteome Discoverer 2.2.1 software as previously described (Yuan et al. 2022).

### MALDI-TOF MS analysis of xanthan lyase

MALDI-TOF MS analyses were performed using a Bruker Microflex LRF MALDI-TOF mass spectrometer (Bruker Daltonics; Billerica, MA, USA) equipped with a nitrogen laser (337 nm) under the control of FlexControl software (version 3.0; Bruker Daltonics; Billerica, MA, USA). Mass spectra were manually collected in positive linear mode within a mass range from 100 to 150 kDa. Ion source voltages 1 and 2 were set at 20 and 18.15 kV, respectively. The lens voltage was set to 9.05 kV. Each spectrum was obtained by the accumulation of 200 laser shots in 100 shot increments.

### Sequence analysis and homology modeling

Sequence alignment among *Ec*Xly and other proteins with high sequence identities was performed by Clustal Omega (https://www.ebi.ac.uk/Tools/msa/clustalo/) and the result was visualized with online software ESPript (https://espript.ibcp.fr/ESPript/cgi-bin/ESPript.cgi). The three-dimensional structure of *Ec*Xly was predicted by utilizing the I-TASSER server (https://zhanglab.ccmb.med.umich.edu/I -TASSER/) for homology modeling (Roy et al. 2010). The predicted three-dimensional structure of the protein was examined and shown using PyMOL software. Developmental tree mapping and analysis were performed using MEGA 7.0 (Kumar et al. 2016).

### Construction of *Ec*Xly deglycosylation mutants

In order to probe the effect of N-glycosylation on enzymatic properties of *Ec*Xly, the glycosylation site N599 was then, respectively, mutated to alanine (A) and aspartic acid (D) and glycine (G) to obtain deglycosylation mutants by RF-cloning using pET-*EcXly* as template. The primers for construction of mutants are given in Additional file 1: Table S1. The deglycosylated mutants were constructed using the same method for the construction of pET-*EcXly* mentioned above. The verified plasmids were then transformed into electrocompetent *E. coli* Rosetta-gami (DE3) pLysS and expressed. The recombinant proteins *Ec*Xly^N599A^, *Ec*Xly^N599D^ and *Ec*Xly^N599G^ were purified as described above for the *Ec*Xly.

### Characterization of *Ec*Xly and its mutants

To determine the specific activity of the xanthan lyase, 1 mg/mL xanthan was incubated with 0.1 mg/mL *Ec*Xly or its mutants in phosphate buffer (pH 6.0). The enzymatic reaction was performed at 30 °C for 20 min, and was ended immediately by heating in boiling water for 5 min. The denatured protein was removed by centrifuging at 12,000 g for 5 min. The supernatant absorbance was measured at 235 nm due to the formation of the double bond between glucuronic acid and terminal mannose of side chain. The definition of a unit of enzyme activity is the amount of enzyme required to increase 1.0 absorbance per minute at 235 nm.

The optimum temperature was assayed by incubating the enzyme–xanthan mixture at various temperatures (25–55 °C, with 5 °C intervals) for 20 min as described above. For the thermostability of xanthan lyase, the proteins were incubated at different temperatures (20–70 °C, with 5 °C intervals) for 2 h, then the reaction was conducted as mentioned above.

For the optimum pH for the enzymatic reaction, the enzyme–substrate mixture was incubated in various buffers (pH 4.0–9.0) at 30 °C for 20 min. To determine the pH stability of proteins, the wild type *Ec*Xly and its mutants were incubated in various buffers (pH 4.0–9.0) at 30 °C for 12 h, respectively, and the residual enzyme activity was tested with the same method described above. The different pH buffers contained citric acid–sodium citrate buffer (pH 4.0–6.0), Na_2_HPO_4_–NaH_2_PO_4_ buffer (pH 6.0–8.0), Tris–HCl buffer (pH 8.0–9.0).

### Determination of kinetic parameter

To investigate the kinetic parameters (*K*_m_ and *V*_max_), the wild type *Ec*Xly and its mutants were incubated with different concentrations of xanthan (0.6, 0.8, 1, 1.2, 1.5, 2, 2.5, 3, 4, 5 mg/mL) for 20 min under the optimum conditions, respectively. Perkin Elmer Lambda 35 UV/VIS Spectrometer/PTP Peltier Temperature Programmer (PerkinElmer, Shanghai, China) was used to monitor *V*_max_. Due to the formation of unsaturated glucuronic acid (the extinction coefficient is 6150 M^−1^ cm^−1^), the molar concentration of the products was transformed by the Lambert–Beer law (Stender et al. 2019). Finally, according to the time course for the formation of unsaturated glucuronic acid, the Michaelis–Menten equation was applied for the calculation of *K*_m_ and *V*_max_ of the wild type *Ec*Xly and its mutants.

### Circular dichroism spectra

Circular dichroism is used to analyze the secondary structure of proteins through the circular dichroism of proteins and the different absorption of left and right circularly polarized light by asymmetric molecules. To monitor the secondary structure of *Ec*Xly and its mutants, circular dichroism (CD) spectra were performed using a JASCO J-815 CD spectrometer (JASCO, Tokyo, Japan). The sample, with a final concentration of 0.2 mg/mL, was prepared in 20 mM Na_2_HPO_4_–NaH_2_PO_4_ buffer (pH 6.0). The data were recorded from 200 to 260 nm at 1 nm intervals at room temperature using a quartz cuvette with 1 mm path length. The value of scan speed and response time was 500 nm/min and 1.0 s, respectively. The CD data were submitted to BeStSel (Beta Structure Selection) online server (http://bestsel.elte.hu.) to analyze the relative proportion of secondary structure (Micsonai et al. 2018).

### Fluorescence spectra

Fluorescence spectroscopy is used to study the spatial conformation of proteins by the characteristics that the side chain groups of aromatic amino acid residues in proteins absorb the incident light in the ultraviolet region and emit fluorescence. Fluorescence spectrometer (F-4600, Hitachi, Japan) was used to characterize the change of tertiary structure of *Ec*Xly and its mutants. The sample was prepared in Na_2_HPO_4_–NaH_2_PO_4_ buffer with a final concentration of 0.05 mg/mL. The fluorescence spectra from emission wavelength of 300 nm to 400 nm were recorded under an excitation wavelength of 280 nm. Each sample was tested three times.

### Statistical analysis

All tests and determinations were carried out in triplicate unless otherwise stated. Data were expressed as the average values ± standard deviation. The significant difference (*P* < 0.05) was analyzed by the Statistical software SPSS 11 (SPSS Inc, Chicago, USA).

## Results and discussion

### Identification of the N-glycosylation in xanthan lyase expressed in *E.coli*

**Fig. 2:**
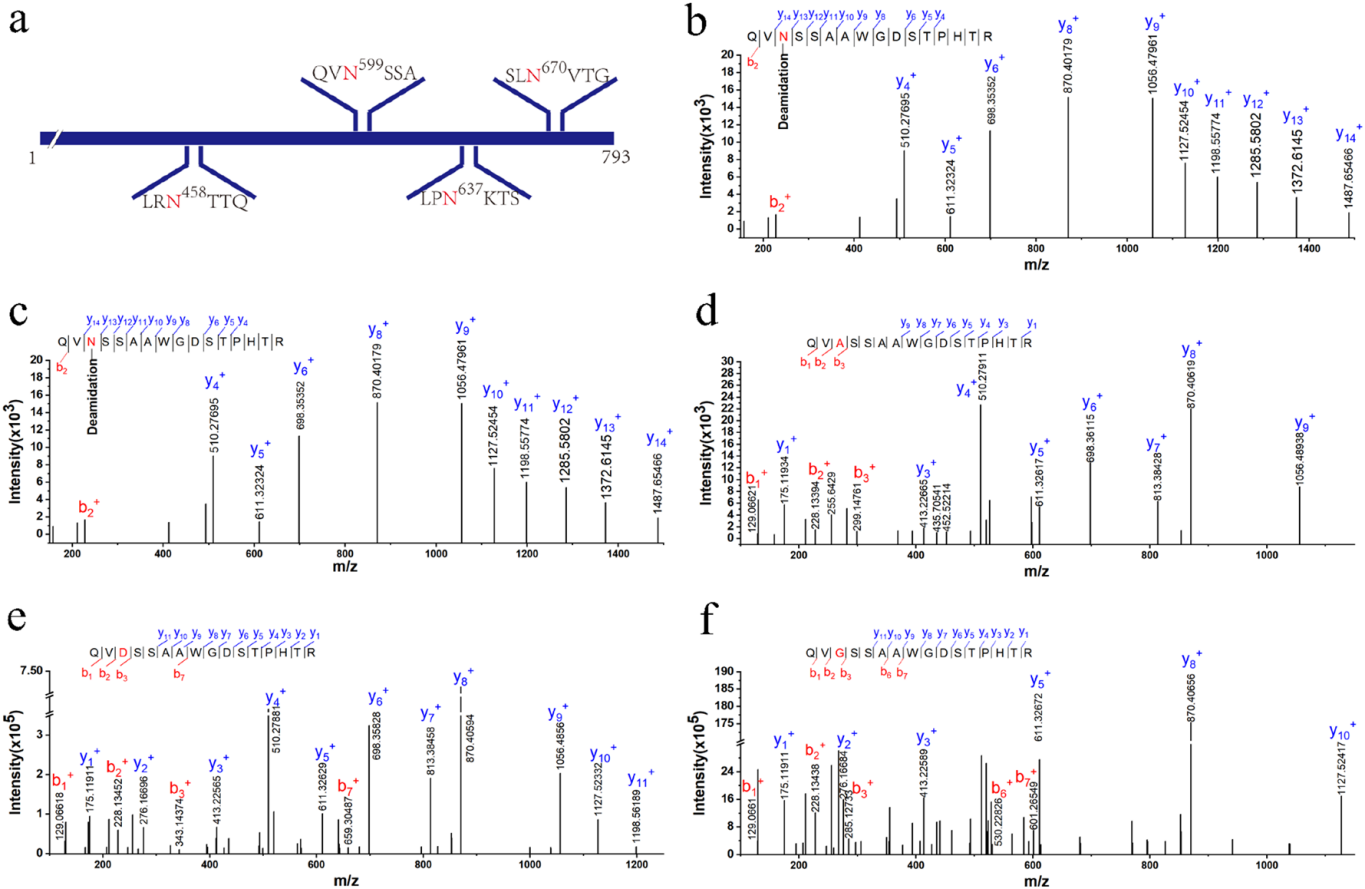
The prediction and analysis of glycosylation sites in *Ec*Xly. **a** The schematic diagram of the 4 potential glycosylation sites in *Ec*Xly. The predicted N-glycosylation sites are shown in red letters. **b**–**f** A mass spectrum of the deglycosylated peptide from *Ec*Xly, *Mi*Xly, *Ec*Xly^N599A^, *Ec*Xly^N599D^ and *Ec*Xly^N599G^, respectively

Four potential N-glycosylation sites in *Ec*Xly were predicted using NetNGlyc 1.0 Server (http://www.cbs.dtu.dk/services/NetNGlyc/) (Fig. 2a). To further confirm the prediction, the purified *Ec*Xly (Additional file 1: Fig. S1) treated with PNGase F, was subjected to mass analysis on the LC–MS/MS. As shown in Fig. 2b, the mass difference of 115 Da between m/z = 1487.65466 (y_14_ peptide) and m/z = 1372.6145 (y_13_ peptide) implied the appearance of deamidation on Asn599 with 0.98 Da mass. The result indicated that N-glycosylation modification only occurred at N599 within the peptide QVN^599^SSA of the enzyme *Ec*Xly. However, the glycosylation modification in *Mi*Xly was not detected (Fig. 2c). This might be due to the different mechanisms of glycosylation modification in different strains (Schäffer and Messner. 2017). It has been previously determined that the specific activity of *Mi*Xly was 28.2 U/mg (Yang et al. 2014), which is significantly lower than that of *Ec*Xly (53.8 U/mg). Therefore, it was hypothesized that the discrepancy in the catalytic capacity of xanthan lyase expressed in different hosts might be caused by the degree of glycosylation.

### Sequence and structural analysis of N-glycosylated *Ec*Xly

**Fig. 3:**
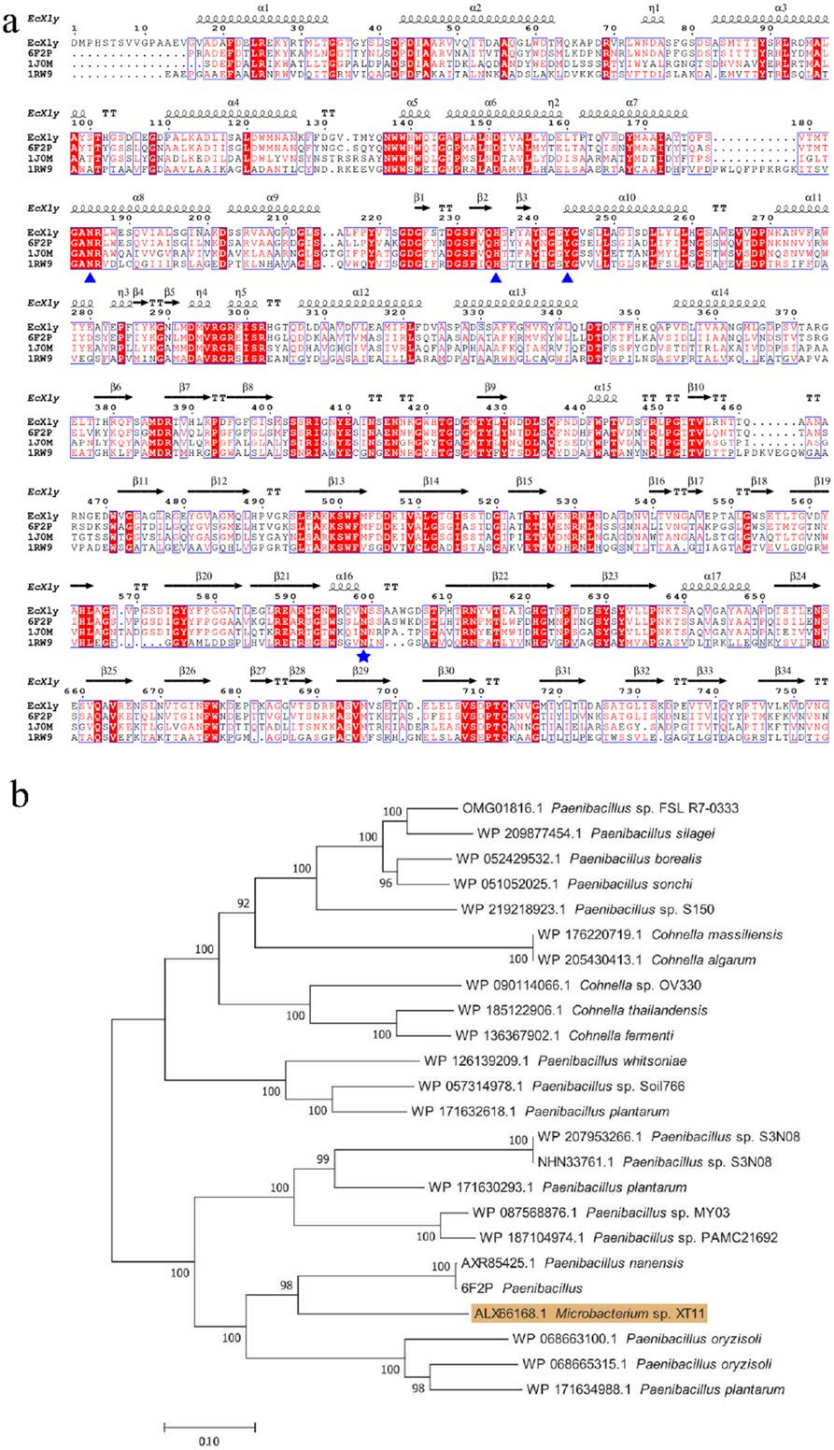
Sequence, structure and phylogenetic tree analyses of *Ec*Xly. **a** Alignment of amino acid sequences of *Ec*Xly, 6F2P, 1J0M, and 1RW9. The amino acid residues that are conserved in all four proteins, are denoted with white letters and highlighted in red. The amino acid residues that are conserved in two or three different proteins are denoted with red letters. The catalytic active site N^185^, H^235^ and Y^244^ was labeled as a blue triangle below the residues, and the glycosylation site N^599^ was indicated by blue star. **b** Phylogenetic tree of *Ec*Xly using MEGA 7.0. Bootstrap values are indicated at branching points

**Fig. 4:**
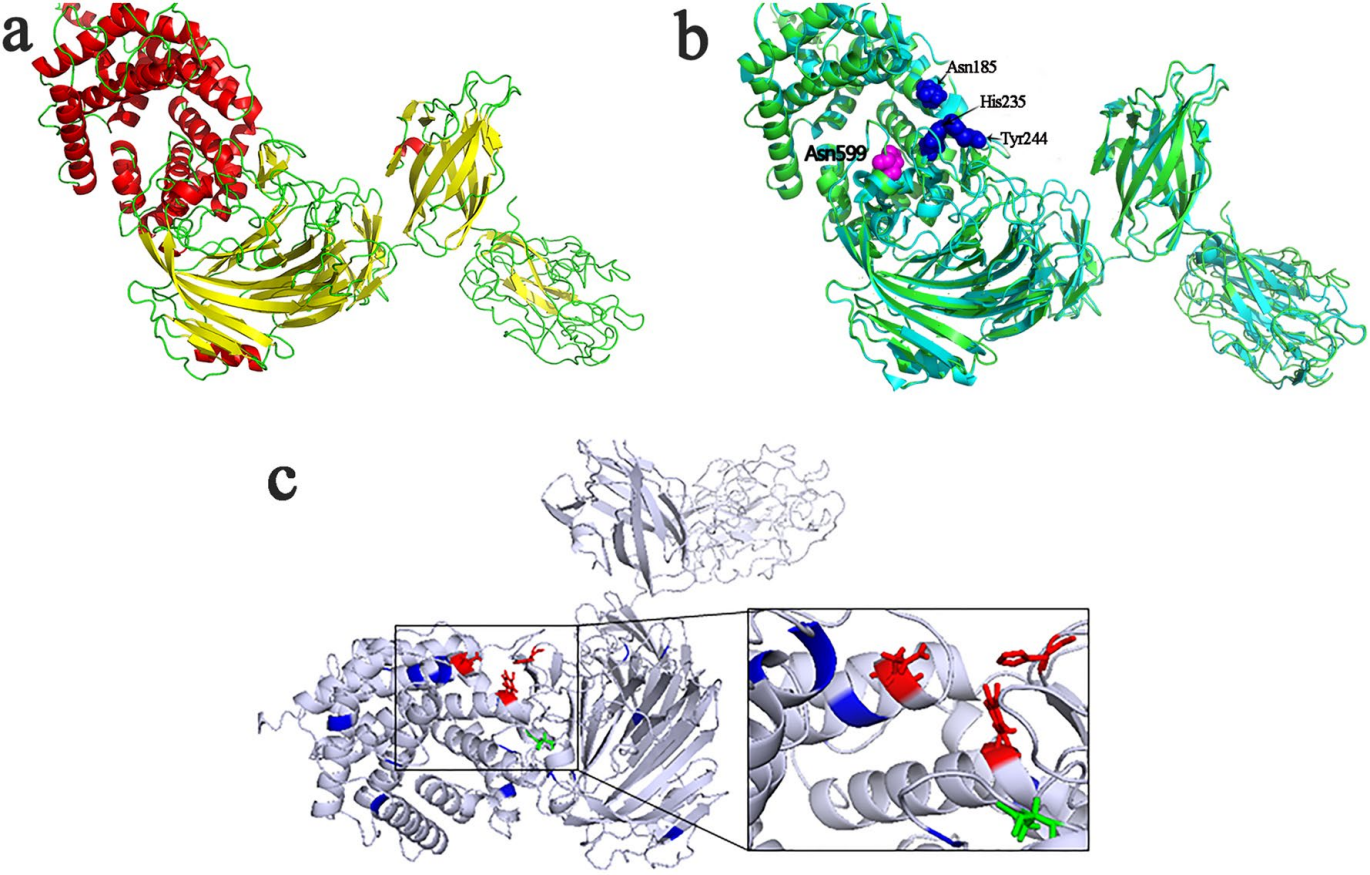
Three-dimensional structure of *Ec*Xly visualized by PyMOL. **a** Secondary structures of *Ec*Xly: α-helix (red); β-sheet (yellow); random coil (green). **b** The structural alignment between *Ec*Xly (green) and PXL (cyan). The catalytic residues were shown in blue sphere and the glycosylation site was shown in magenta sphere. **c** Structural elements around the active sites. Tryptophan residues were colored in blue. The catalytic residues were colored in red rod. The glycosylation site N599 was highlighted in green rod. The picture in the black box was a close-up view of the catalytic residues of *Ec*Xly

Sequence alignment revealed high sequence identity of *Ec*Xly with xanthan lyase from *Paenibacillus nanensis* (PXL) (PDB: 6F2P, 69.45% identity) (Jensen et al. 2019), *Bacillus* sp. GL1 xanthan lyase (PDB: 1J0M, 50.4% identity) (Hashimoto et al. 2003) and chondroitin AC lyase (PDB: 1RW9, 35.16% identity) (Lunin et al. 2004). Three conserved sites including N^185^, H^235^ and Y^244^ might be the catalytic residues in *Ec*Xly (Fig. 3a) (Jensen et al. 2019). Meanwhile, the glycosylated residue N599 was conserved. The phylogenetic analysis showed that the *Ec*Xly fell in a same cluster with PXL (Fig. 3b). Subsequently, the three-dimensional structure of *Ec*Xly was predicted and visualized (Fig. 4a). The TM-scores of 6F2P, 1J0M and 1RW9 for *Ec*Xly were 0.875, 0.621 and 0.601, respectively, and the bond length root mean square deviations were 0.75 Å, 1.57 Å, and 2.21 Å, respectively. Moreover, the structural superposition between *Ec*Xly and PXL (Fig. 4b) revealed that the structural characteristic of *Ec*Xly was similar to other polysaccharide lyases of the PL8 family. The catalytic domain (Asp^1^-Ile^719^) of *Ec*Xly contained α-helix, β-sheet and random coil secondary structure, and the carbohydrate binding domain (Glu^784^-Glu^1146^) contained β-sheet and random coil secondary structure. As shown in Fig. 3a and Fig. 4b, the glycosylation site N599 was located in the catalytic domain and in close proximity to the catalytic residues. Therefore, these results provide support for that glycosylation site (N599) may play an important role in enzymatic properties of xanthan lyase.

### Basic characterizations of N-glycosylation xanthan lyase

To confirm the influence of N-glycosylation on basic characterizations of *Ec*Xly, deglycosylation was performed by substituting the glycosylation site N599, respectively, with alanine (uncharged and non-polar R group), aspartic acid (charged and polar R group) and glycine according to previous researches (Kim et al. 2012). The *Ec*Xly^N599A^, *Ec*Xly^N599D^ and *Ec*Xly^N599G^ mutants were obtained by Ni–NTA affinity purification after expression at 16 °C (Additional file 1: Fig. S1). The glycosylation modification of *Ec*Xly^N599A^, *Ec*Xly^N599D^ and *Ec*Xly^N599G^ mutants was not detected (Additional file 1: Fig. 2d–f). Usually, the absence of the N-glycan chain led to the decrease of molecular weight (Amore et al. 2017). The molecular weight of *Ec*Xly (m/z: 134866.633[M + H]^+^) is higher than that of the mutant *Ec*XlyN^599D^ (m/z: 133837.739[M + H]^+^) (Additional file 1: Fig. S2), it could be speculated that the glycosylation site of *Ec*Xly was probably linked to short chain sugars.

**Fig. 5:**
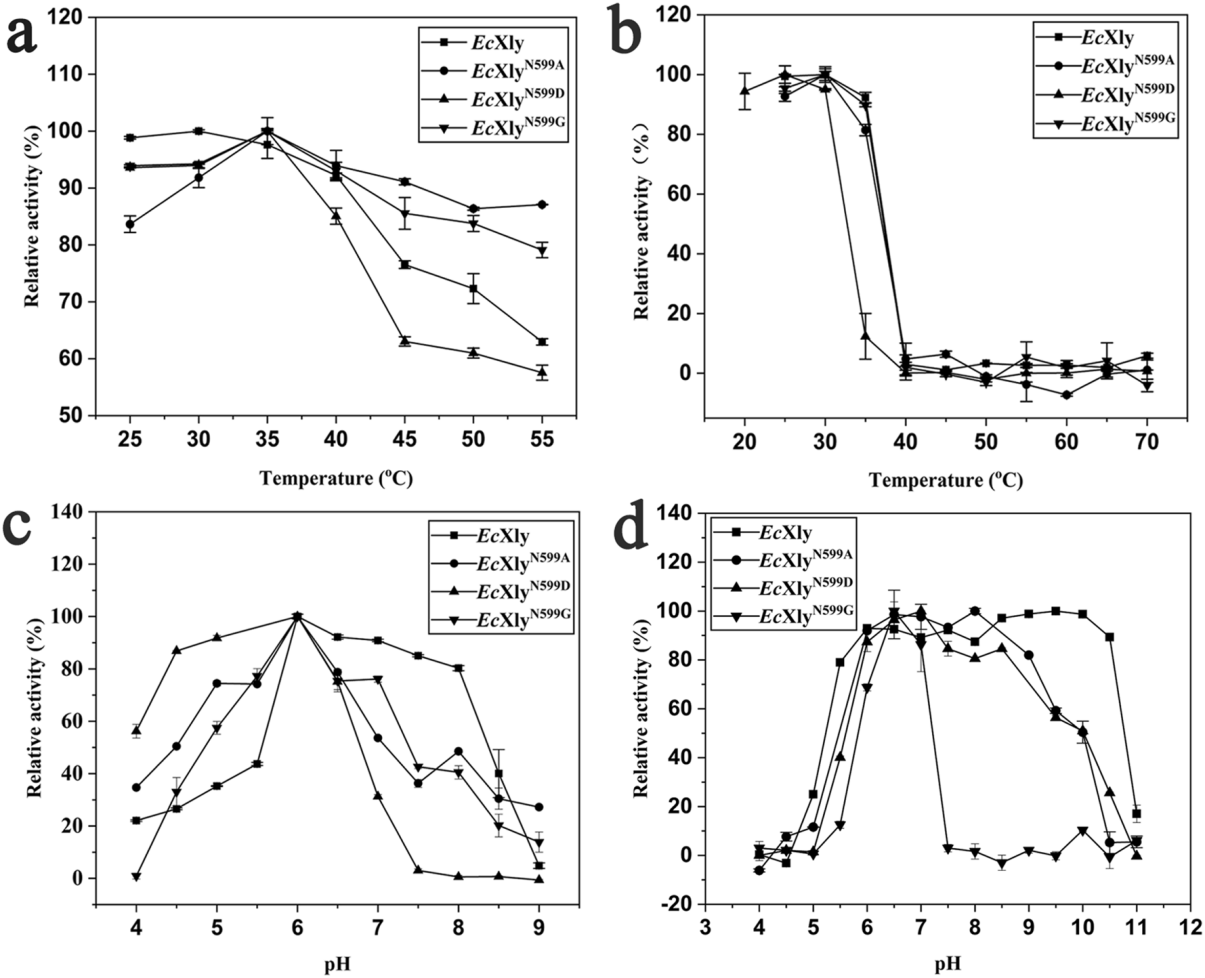
Biochemical characteristics of *Ec*Xly and its mutants. **a** The optimum temperature, **b** thermal stability, **c** optimum pH and **d** pH stability

The effect of N-glycosylation on the temperature optimum and thermostability of *Ec*Xly was examined. As shown in Fig. 5a, the optimum temperature of the *Ec*Xly was lower than that of *Mi*Xly (Yang et al. 2014). Consistent with this result, when the sugar chain was removed from *Ec*Xly through site-directed mutagenesis, higher optimum temperatures of the mutants were detected. All these results suggested that glycosylation in N599 had significant influence on the optimum temperature of xanthan lyase. This investigation was consistent with previous reports that the glycosylated xylanase displayed a decreased optimum catalytic temperature compared with the unglycosylated enzyme (Fonseca-Maldonado et al. 2013).

Some studies had indicated that N-glycosylation was closely related to the thermal stability of the cellulase or cellobiohydrolase (Amore et al. 2017; Qi et al. 2014). However, as shown in Fig. 5b, no significant difference was observed in the thermal stability between *Ec*Xly and *Mi*Xly (Yang et al. 2014). Moreover, both *Ec*Xly and its mutants *Ec*Xly^N599A^ and *Ec*Xly^N599G^ were relatively stable at temperatures ranging from 20 °C to 35 °C, as high residual activities were observed even those samples were incubated at selected temperatures for 2 h. Compared with other samples, *Ec*Xly^N599D^ was relatively stable at temperatures ranging from 20 °C to 30 °C. The thermal stability of *Ec*Xly^N599D^ sharply decreased, which might be due to the changes in the local secondary structure of *Ec*Xly^N599D^ (Niu et al. 2015). The results indicated that glycosylation had no effect on the thermal stability of xanthan lyase. Similar results were also found in the study of Li et al. (2018), who observed that the glycosylation of alginate lyase had no impact on its thermal stability.

As shown in Fig. 5c, d, effects of glycosylation on pH optimum and stability of *Ec*Xly were examined. Both *Ec*Xly and *Mi*Xly obtained maximum activity at pH 6.0 (Yang et al. 2014). When the *Ec*Xly was deglycosylated (*Ec*Xly^N599A^, *Ec*Xly^N599D^, and *Ec*Xly^N599G^), no significant changes in the pH optimum were detected. The result demonstrated that the glycosylation had no effect on the optimum pH of xanthan lyase. The results are concordant with the observation that both the glycosylated and deglycosylated bovine enterokinase light chain reached their maximum activity at pH8.0 (Wang et al. 2018).

The previous study showed that *Mi*Xly could maintain high enzyme activity in an alkaline environment (pH value ranging from 5.5 to 10.5) for 2 h (Yang et al. 2014). A similar pH stability profile was obtained for *Ec*Xly when it was maintained for 12 h (Fig. 5d), which indicated that glycosylated *Ec*Xly might have higher tolerance to alkaline conditions. Compared to *Ec*Xly, the enzyme activities of the mutants *Ec*Xly^N599A^ and *Ec*Xly^N599D^ decreased when the pH value exceeded 9.0, while *Ec*Xly^N599G^ decreased sharply at pH 7.0, which suggested that removal of the sugar chain decreased the tolerance of xanthan lyase against the alkaline environment. The possible reason for the substantially reduced pH stability of the mutants was well described by Solá and Griebenow. (2009), who found that absence of glycan increased the solvent accessible surface area of the protein, resulting in reducing internal electrostatic interaction. A recent study has shown that the protein with weak hydrophobic interaction among amino acid had worse pH stability (Liu et al. 2021). As an uncharged amino acid, glycine had little electrostatic interaction with surrounding amino acids, and its hydrophobic interaction was also very weak compared with uncharged alanine. This finally led to the lowest pH stability of *Ec*Xly^N599G^. All the above investigations suggested that the N-glycosylation in N599 has a strong impact on basic characterizations of xanthan lyase.

### The role of N-glycosylation in the catalytic performance of xanthan lyase

Under the optimal condition, the kinetic parameters of *Ec*Xly and its mutants were determined using xanthan as substrate (Table 1). The *K*_m_ value of *Ec*Xly was higher than that of the *Mi*Xly (Yang et al. 2014). Compared with the *Ec*Xly, the *K*_m_ values of all the mutants significantly decreased, which demonstrated that the glycan chain may hinder the affinity of the protein to the substrate (Kołaczkowski et al. 2020). A similar result was reported by Wei et al. (2013), who found that the *K*_m_ value of β-glucosidase expressed in *P. pastoris* after deglycosylation was lower than that of the wild type. Among the mutants, *Ec*Xly^N599A^ and *Ec*Xly^N599G^ had a higher affinity, which may be due to increasing hydrogen bonds formed between the substrate and the alanine or glycine (Zheng et al. 2018). It should be noted that the increased substrate affinity of mutants compensated for the reduced *K*_cat_, resulting in no decrease in catalytic efficiency (*K*_cat_/*K*_m_) compared to the *Ec*Xly. These results indicated that deglycosylation could improve the substrate affinity, but had no effect on catalytic efficiency.

**Table 1:** The kinetic parameters of *Mi*Xly*, Ec*Xly and its mutants (*Ec*Xly^N599A^, *Ec*Xly^N599D^ and *Ec*Xly^N599G^)

Enzyme	*K*_m_(μM)	*K*_cat_(min^−1^)	*K*_cat_/*K*_m_(μM^−1^ min^−1^)
*Mi*Xly^d^	38.455	0.181	0.031
*Ec*Xly	18.752 ± 1.042^a^	0.254 ± 0.016^a^	0.014 ± 0.001^a^
*Ec*Xly^N599A^	1.361 ± 0.267^c^	0.021 ± 0.003^c^	0.016 ± 0.003^a^
*Ec*Xly^N599D^	8.414 ± 0.910^b^	0.109 ± 0.009^b^	0.013 ± 0.001^a^
*Ec*Xly^N599G^	1.314 ± 0.016^c^	0.021 ± 0^c^	0.015 ± 0^a^

^a,b,c^The superscript different letters in the same column represent significant difference (*P* < 0.05), whereas the superscript same letter in the same column suggests no significant difference (*P* > 0.05)

^d^ The kinetic data were from Yang et al. (2014)

**Fig. 6:**
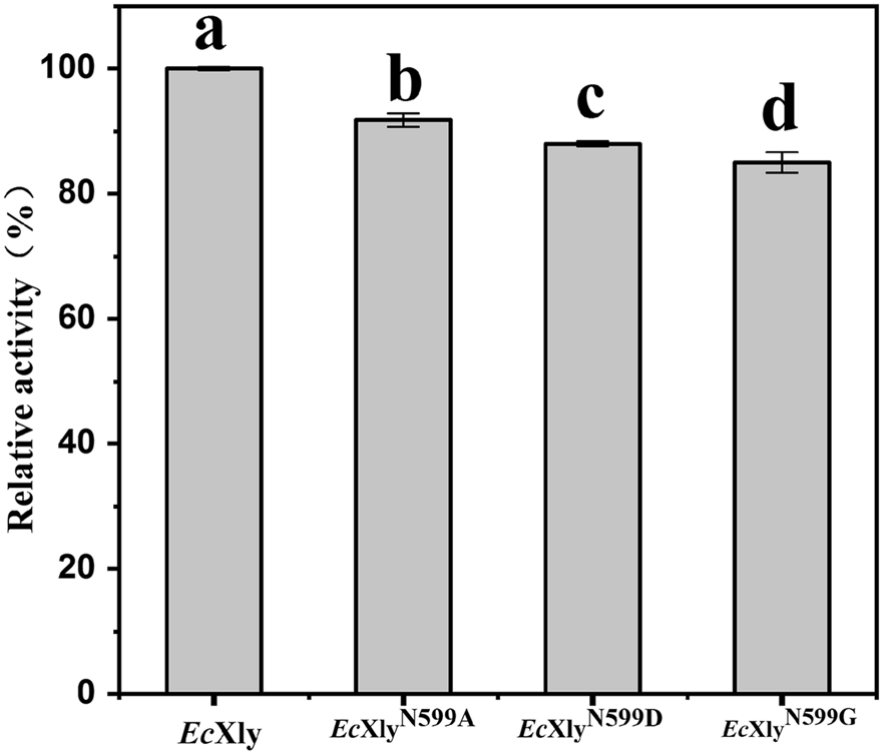
The comparison of enzyme activity between *Ec*Xly and its mutants. The data represented the averages ± standard deviations. Each experiment was repeated three times. The significant difference was indicated by different letters (*P* < 0.05)

Furthermore, the enzyme activities of *Ec*Xly and its mutants were analyzed (Fig. 6, Table 1). The enzyme activity of *Ec*Xly was 53.8 U/mg, which was higher than that of *Mi*Xly (28.2 U/mg) (Yang et al. 2014). Compared with wild type *Ec*Xly, the enzyme activities of the *Ec*Xly^N599A^, *Ec*Xly^N599D^ and *Ec*Xly^N599G^ decreased by 10%, 12% and 15%, respectively. The reduced enzyme activity might be due to the lack of sugar chain at N599 after mutation, which leads to the structural changes of the enzyme around the catalytic residues (Varki 2017). The results were in agreement with the demonstration of Gusakov et al. (2017) that the removal of glycan in N395 located on catalytic domain decreased the enzyme activity of cellobiohydrolase. All these investigations showed that N-glycosylation plays a key role in the catalytic performance of *Ec*Xly.

### The mechanism of N-glycosylation affecting the properties of xanthan lyase

**Fig. 7:**
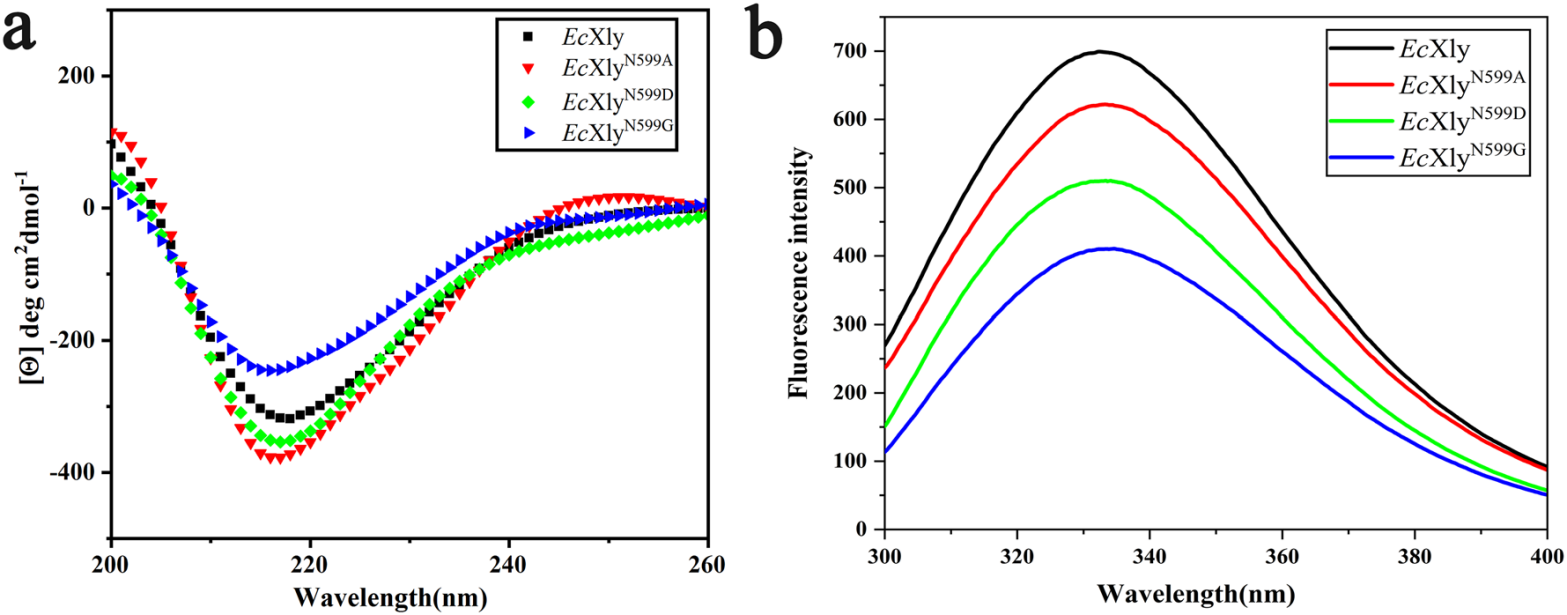
The structural analysis of *Ec*Xly and its mutants (*Ec*Xly^N599A^, *Ec*Xly^N599D^ and *Ec*Xly^N599G^). **a** Far-UV CD spectra. **b** The fluorescence spectra

To further clarify the possible mechanism that N-glycosylation affects properties of xanthan lyase, the secondary and tertiary structures of *Ec*Xly and its mutants were analyzed by far-UV spectrum and fluorescence spectroscopy. The result indicated that *Ec*Xly and its mutants exhibited a similar profile (Fig. 7a), with a negative peak occurring around 216 nm, indicating that *Ec*Xly was a protein rich in β-sheets (Fig. 4a) (Chen et al. 2021). Quantitative analysis of secondary structure (Table 2) also suggested that the secondary proportion of deglycosylated *Ec*Xly seemed to have little changed, excepted the *Ec*Xly^N599D^. It was found that the α-helix content of *Ec*Xly^N599D^ increased as the β-sheet content decreased. The reason might be that aspartic acid was prone to form hydrogen bonds with surrounding amino acids to promote the formation of α-helices (Ramirez et al. 2017). The poor thermal stability of *Ec*Xly^N599D^ may be due to the decreased β-sheet content, which resulted in a more flexible local structure of the protein. A similar result was reported by Niu et al. (2015). All these results revealed that N-glycosylation had no effect on the secondary structure of xanthan lyase, which is in accordance with the previous works (Wang et al. 2018).

**Table 2:** The secondary structure proportion of *Ec*Xly and its mutants (*Ec*Xly^N599A^, *Ec*Xly^N599D^ and *Ec*Xly^N599G^)

Enzyme	α-helix(%)	β-strand(%)	Turn(%)	Others(%)
*Ec*Xly	3.8	41.6	12.4	42.4
*Ec*Xly^N599A^	3.3	44.4	12.6	39.8
*Ec*Xly^N599D^	5.9	37.5	12.8	43.8
*Ec*Xly^N599G^	4.5	41.8	12.3	41.4

The fluorescent spectra of *Ec*Xly and its mutant were used to determine their tertiary structure (Fig. 7b). There were approximately 20 tryptophan residues with intrinsic fluorescence in *Ec*Xly (Fig. 4c). Therefore, the tertiary structure of *Ec*Xly was reflected by recording the emission spectrum of tryptophan excited at 280 nm. According to the fluorescence spectra, the fluorescence intensity of *Ec*Xly^N599A^, *Ec*Xly^N599D^ and *Ec*Xly^N599G^ decreased. It was suggested that the mutation at glycosylation site changed the microenvironment of tryptophan residue and had an effect on the overall tertiary structure of xanthan lyase (Zhao et al. 2020). Different from the non-polar amino acid alanine, the polar amino acid aspartic acid and glycine might have a greater impact on the microenvironment of tryptophan residues, leading to significant changes in fluorescence intensity (Ghisaidoobe and Chung. 2014). To further confirm the fluorescent spectra investigation, structural elements around the active sites of xanthan lyase were predicted. As shown in Fig. 4c, the glycosylation site, catalytic residues and some tryptophan residues were very close with each other, which probably lead to the significant influence of the sugar chain on the microenvironment of catalytic residues (Boer and Koivula. 2003) and subsequently caused the changes in enzyme properties (Miller and Brambley. 2020) (Fig. 6).

## Conclusions

In conclusion, the xanthan lyase heterologously expressed in *E. coli* was modified by N-glycosylation at N599. Our work revealed that the glycosylation site was located in the catalytic domain and close to the key catalytic residues, which changed the microenvironment of tryptophan residue and affected the overall tertiary structure of xanthan lyase. The N-glycosylation modification played an important role in the basic characterizations and catalytic performance of xanthan lyase through adjusting its structure, including changing the optimum temperature, improving the tolerance to alkaline conditions and the specific activity, and decreasing the substrate affinity of xanthan lyase. Overall, the findings of this research provide valuable insights for the rational design of xanthan lyase with better catalytic performance. Furthermore, future works should be extended to broad the application of N-glycosylated xanthan lyase and the industrial production of modified xanthan with excellent physicochemical and physiological functions.

### Supplementary Information

**Additional file 1**: **Table S1** Scheme for site-directed mutagenesis of pET-*Ec*Xly plasmids. **Fig. S1** The SDS-PAGE analysis of purified *Ec*Xly and its mutants (*Ec*Xly^N599A^, *Ec*Xly^N599D^ and *Ec*Xly^N599G^). Lane 1-4 was the purified *Ec*Xly,* Ec*Xly^N599A^, *Ec*Xly^N599D^ and *Ec*Xly^N599G^, respectively. Lane M was the protein High Maker. **Fig. S2** Molecular mass analysis of *Ec*Xly (a) and* Ec*Xly^N599D^ (b) using MALDI-TOF MS.

## Data Availability

All data produced or analyzed for this study are included in the published article and its additional information files.
