# Supplementary material for: Dissecting the essential role of N-glycosylation in catalytic performance of xanthan lyase
Source: Bioresour Bioprocess. 2022 Dec 16;9(1):129. doi: 10.1186/s40643-022-00620-5 (PMC10992191; doi:10.1186/s40643-022-00620-5)
Supplement: Supplementary file 1 — Additional file 1: Table S1 Scheme for site-directed mutagenesis of pET-EcXly plasmids. Fig. S1 The SDS-PAGE analysis of purified EcXly and its mutants (EcXlyN599A, EcXlyN599D and EcXlyN599G). Lane 1-4 was the purified EcXly, EcXlyN599A, EcXlyN599D and EcXlyN599G, respectively. Lane M was the protein High Maker. Fig. S2 Molecular mass analysis of EcXly (a) and EcXlyN599D (b) using MALDI-TOF MS. [file 40643_2022_620_MOESM1_ESM.docx]

**Additional file 1**

**Dissecting the essential role of N-glycosylation in catalytic performance of xanthan lyase**

Jingjing Zhao^1#^, Qian Wang^1,2#^, Xin Ni^1^, Shaonian Shen^1^, Chenchen Nan^1^, Xianzhen Li^1^, Xiaoyi Chen^1^* and Fan Yang^1^*

^1^ School of Biological Engineering, Dalian Polytechnic University, Ganjingziqu, Dalian 116034, People’s Republic of China

^2^ Division of Biotechnology, Dalian Institute of Chemical Physics Chinese Academy of Sciences, Dalian 116023, People’s Republic of China

* Correspondence: chen-xy@dlpu.edu.cn (XC) or yang_fan@dlpu.edu.cn (FY)

^1^ School of Biological Engineering, Dalian Polytechnic University, Ganjingziqu, Dalian 116034, People’s Republic of China

^#^ Equal contributors

**Table S1** Scheme for site-directed mutagenesis of pET-*EcXly* plasmids.

| Strains^a^ | Resulting plasmids | | Oligonucleotide (Sequence, 5’-3’)^b^ |
| --- | --- | --- | --- |
| *Ec*Xly | | pET- *EcXly* | Fwd(GACGACAAGGCCATGGCTGATATCATGGAC-  ATGCCTCACAGCACGAGCGTC) |
|  |  |  | Rev(CGGGCTTTGTTAGCAGCCGGATCTCTCGATG-  GCGGAGACCAGCTGAAT) |
| *Ec*Xly^N599A^ | | pET-*EcXly*^N599A^ | N599A-rev( GCGGCGGAGGACGCCACCTGCCTC) |
| *Ec*Xly^N599D^ | | pET-*EcXly*^N599D^ | N599D-rev(CGGAGGAGTCCACCTGCCTCCAG ) |
| *Ec*Xly^N599G^ | | pET-*EcXly*^N599G^ | N599G-rev(GCGGAGGAGCCCACCTGCCTCCAG) |

^a^ The corresponding plasmid was transformed into *E. coli* Rossetta-gami(DE3)pLysS.

^b^ The appropriate mutations in primers are underlined.


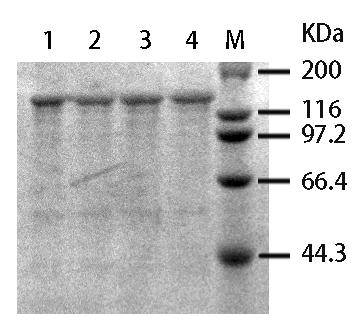


**Fig. S1** The SDS-PAGE analysis of purified *Ec*Xly and its mutants (*Ec*Xly^N599A^, *Ec*Xly^N599D^ and *Ec*Xly^N599G^). Lane 1-4 was the purified *Ec*Xly, *Ec*Xly^N599A^, *Ec*Xly^N599D^ and *Ec*Xly^N599G^, respectively. Lane M was the protein High Maker


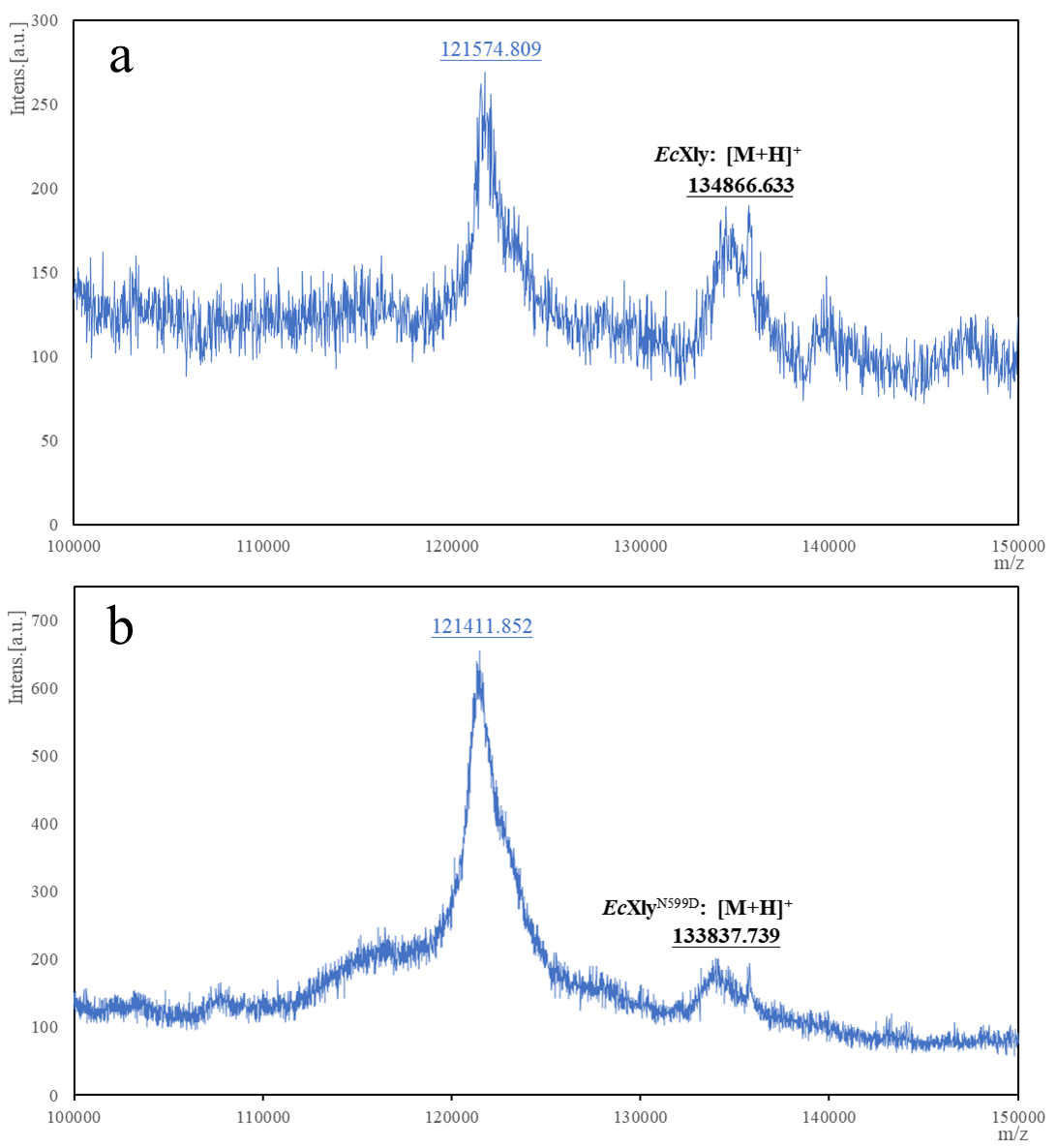


**Fig. S2** Molecular mass analysis of *Ec*Xly (a) and *Ec*Xly^N599D^ (b) using MALDI-TOF MS
